# Demographics of co-ageing complex systems: from infected worms to chess games

**DOI:** 10.1098/rsos.240932

**Published:** 2024-11-13

**Authors:** Cagatay Eskin, Dervis Can Vural

**Affiliations:** ^1^ Department of Physics and Astronomy, University of Notre Dame, Notre Dame, IN, USA

**Keywords:** complex systems, ageing, demographics, ecology, co-ageing, Networks

## Abstract

Ageing, as defined in terms of the slope of the probability of death versus time (hazard curve), is a generic phenomenon observed in nearly all complex systems. Theoretical models of ageing predict hazard curves that monotonically increase in time, in discrepancy with the peculiar ups and downs observed empirically. Here we introduce the concept of co-ageing, where the demographic trajectories of multiple cohorts couple together, and show that co-ageing dynamics can account for the anomalous hazard curves exhibited by some species. In our model, multiple interdependency networks inflict damage on one other proportional to their number of functional nodes. We then fit our model predictions to three datasets describing (i) co-ageing worm–pathogen populations and (ii) competing tree species. Lastly, we collect data on the mortality statistics of (iii) chess games to demonstrate that co-ageing dynamics is not exclusive to biological systems.

## 1. Introduction

Typically, simple systems consisting of few constituents, such as radioactive nuclei or a sledgehammer, will fall apart with constant probability per unit time. By contrast, the failure probability of a complex system such as a human or a jet engine tends to increase by many folds over its life span. The positive slope of failure probability versus time (called the ‘hazard curve’ or ‘mortality curve’) is the demographic definition of ageing. Radioactive nuclei do not age, whereas jet engines do.

Evolutionary theories of ageing offer a plausible explanation for this positive slope: since an organism risks experiencing an external hazard at any point in its life, it is a better evolutionary strategy to reproduce early on rather than later in life. In such early reproducing lineages, natural selection cannot eliminate late-acting deleterious traits, which manifest as ageing (mutation accumulation theory [1–4]). Some late-acting deleterious genes might even happen to enhance early life success, thereby getting positively selected (antagonistic pleiotropy theory [2,5,6]).

Vural *et al*. [7] argued that ageing is a generic phenomenon characteristic of systems consisting of a large number of interdependent components. Networks of interdependence are fragile: if one component malfunctions, so will others that crucially depend on it. The failure statistics of such networks was shown to accurately describe the demographic trajectories of biological species as well as complex mechanical devices [7]. The interdependence network picture does not negate the evolutionary arguments outlined above but works in tandem and has since been fruitful in progressing our empirical and theoretical understanding of ageing populations [7–16] (see also the appendix of Stroustrup *et al*. [17] for a direct test of the model).

Evolutionary and interdependency network models both predict hazard curves that monotonically increase in time. However, empirically, hazard curves with peculiar ups and downs have been observed, in apparent contradiction with theory [18,19]. Some organisms have a higher probability of death when younger, while others start anti-ageing midlife, only to continue ageing later. How should we interpret and predict such features?

We hypothesize here that a non-monotonic hazard curve can signal a coupling between the ageing processes of multiple species. For example, some tree species in early successional forests exhibit non-monotonic hazard curves during the ‘thinning’ phase of stand development [20]. The primary factor contributing to mortality at this stage is suppression, arising from intense competition for vital resources such as nutrients, water and sunlight [21–23]. Another example is worms experiencing an increase in early life mortality owing to bacterial infections. These infections not only manifest as faster ageing and shorter lifespans but also as qualitatively distinct kinks or bumps in the worm’s hazard curve [24,25].

**Figure 1.**
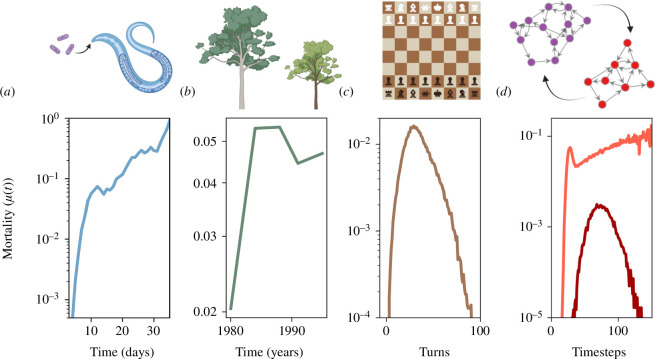
The co-ageing systems we study here, alongside their typical hazard curves. (*a*) *Caenorhabditis elegans* colonized by pathogenic *Escherichia coli*. The worm’s mortality curve exhibits a distinct early life ‘kink’. (*b*) *Acer macrophyllum*, the bigleaf maple, during the thinning phase of its lifecycle, exhibits a similar non-monotonic mortality pattern, as it experiences heightened competition for resources. (*c*) Chess games. Chess defence networks inflict damage on each other similar to biological antagonists, thereby exhibiting similar non-monotonic mortality curves. (*d*) Our theoretical model, where two interdependency networks inflict damage on each other proportional to the number of their functional nodes. The two curves correspond to two different parameter sets.

In this paper, we introduce the concept of ‘co-ageing’ and offer a theoretical model to interpret and predict the demographic data of co-ageing populations. In our model, two fragile interdependency networks assault each other and influence each other’s failure statistics. Then using this model, we analyse four empirical datasets: three from the available literature and one gathered by us (figure 1).

The first dataset [24] measures the hazard curves of *Caenorhabditis elegans* whose digestive tracks are colonized by pathogenic *Escherichia coli*. The immune system of the worm inflicts damage on the colony, and vice versa, leading to an anomalous hazard curve. The second dataset [20] measures the demographic trajectories of three different species of trees from early successional forests. This data span the ‘thinning’ stage, which is known as the dynamic period of stand development in trees. During the thinning stage, one of the most prominent drivers of mortality is inter- and intra-specific competition. We argue here that the unusual hazard curves observed in many tree species are owing to co-ageing, i.e. individuals indirectly damaging each other by cutting off access to vital resources.

One of the appeals of our interdependency picture over its evolutionary counterparts is its applicability beyond biology: just as ageing can be observed in non-biological complex machines [7], we argue here, so can co-ageing. To demonstrate this, as a third study case, we gather the failure statistics of chess games, where, much like the worm–pathogen and tree–tree systems, the players continuously assault each others’ network of defences and exhibit similar co-ageing signals in their demographic trajectories.

We shall see that worms and pathogens, competing trees and chess battles all have similar hazard curves and can all be accurately fitted to our co-ageing interdependency network theory presented here.

Furthermore, our model can account for the difference in hazard curves of infected (co-ageing with bacteria) and antibiotic-fortified (non-co-ageing) worms by simply turning down a single parameter (that quantifies the antagonist-inflicted damage). By altering this one parameter, the model can also account for the difference in hazard curves of chess players with varying skill levels: a higher skill level difference results in a larger impact per piece, thereby quantifying the antagonist-inflicted damage.

## 2. Network model of co-ageing

Our model consists of two complex networks, each, consisting of nodes and directional edges, which we interpret as functional constituents and the interdependency between them.

### 2.1. Initialization of networks (evolutionary time scales)

We begin by creating a large cohort of network pairs representing two adversarial species, A and B, with number of nodes $N_{A}$ and $N_{B}$. All members of a species are assumed to have identical network structures. For large networks, randomizing network connections between individuals yields identical results, with insignificant additional noise (see the electronic supplementary material).

Networks are built by adding one node at a time. To avoid coincidental occurrences of disconnected sub-networks, each newly added node connects inwards randomly to one existing node and outwards to another.

This random growth of interdependency represents a ‘constructive neutral’ evolutionary process, first (qualitatively) hypothesized by Stoltzfus [26]. It was shown earlier [7] that a non-neutral growth (where the connection probability is non-uniform) leads to similar outcomes in mortality statistics; so we do not explore here the effect of topology further.

### 2.2. Ageing of networks (individual time scales)

After growing the networks, as if by constructive neutral evolution [26], we probe their fragility, alone (ageing) and together (co-ageing). During the course of an ageing simulation, every node assumes one of two states: functional or dysfunctional.

We age networks as follows: at each time step, some of the nodes in A will be marked to malfunction. This is done in two rounds. In the first round, each node is marked with probability $d_{A}$. This is the steady damage rate experienced by the organism in the absence of its adversary. We then iteratively ‘propagate’ this damage throughout the network, by also marking the nodes that lost more than half of their dependees.

In the second round, additional nodes are marked for failure with a probability $\alpha_{BA}(t)$ proportional to the fraction of functional nodes $f_{B}/N_{B}$, in the opponent network B, namely, $\alpha_{BA}(t)=C_{A}f_{B}/N_{B}$, where the ‘co-ageing parameter’ $C_{A}$ quantifies the ageing impact of B on A. This damage is also propagated in a similar fashion to round one.

After each round, we also allow for the damaged node to revert to its functional state with probability $r_{A}$. This is the repair rate.

At the end of these two rounds, we update the labels of the marked nodes. If a node was marked for failure during the first round or owing to a majority of its dependees malfunctioning during the first round (even though it might have failed in a second round), we register its cause of death as ‘intrinsic damage’ and otherwise as ‘co-ageing damage’. For the dysfunctional nodes whose dependees were damaged half and half by intrinsic and co-ageing damage: if the node was marked for failure during a first round, we register the cause of death as intrinsic ageing. If marked during a second round, co-ageing.

Then, A’s antagonist B also undergoes damage and repair in an identical fashion, with analogous damage rates $d_{B}$, $\alpha_{AB}(t)$, and repair rate $r_{B}$; and one time step is complete. Then, we move on to the next time step and repeat this process until both of the networks die.

**Figure 2.**
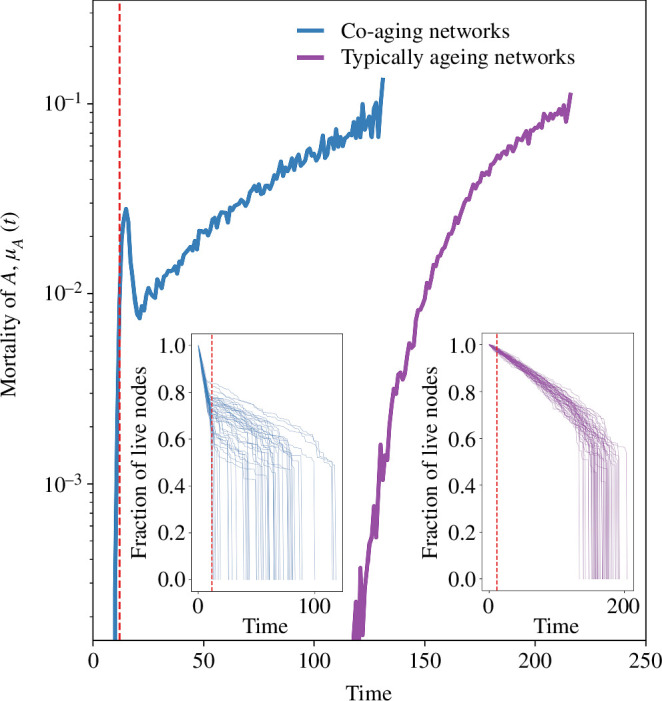
The hazard curves of co-ageing (left) and typically ageing (right) complex networks. A kink or bump is the hallmark of co-ageing and occurs near the mean lifespan of the antagonist (vertical dashed line). The insets show the decline of the function of 50 individual networks, where co-ageing manifests as a sudden reduction of slope coinciding, again, with the mean lifespan of the antagonist. The right curve and right inset describe a network ageing solo, which lacks these peculiar features. Co-ageing parameters are $C_{A}=0.015$ (left) versus $C_{A}=0$ (right), while other parameters (see the electronic supplementary material for values) are kept constant.

We proclaim a network dead if the fraction of its functional nodes goes below 10%. Since the overwhelming majority of networks collapse suddenly once the functional nodes fall below 40–60% (see figure 2, insets), our outcomes do not sensitively depend on the value of this threshold for large networks (see the electronic supplementary material).

Once both networks die, we record their age of death and cause of death and move on to another pair and age them the same way.

**Figure 3.**
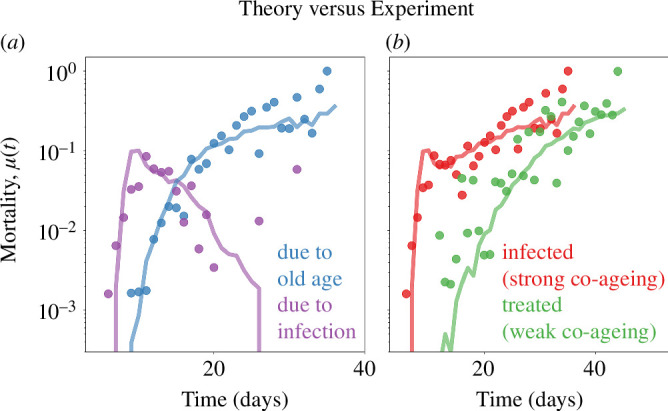
Co-ageing of *C. elegans* and its pathogen *E. coli* (dots), fit to co-ageing networks (curves). (*a*) The decomposition of all-cause hazard curve of the infected worm population into cause-specific hazard curves. As experimental measurements can go beyond counting total deaths and resolve the cause of deaths [24], we have also labelled the cause of deaths of our simulated networks, which agrees well with the experiment. (*b*) All-cause mortality for infected (sum of cause-specific hazards in (*a*)) versus antibiotic-treated worm populations. Modifying the theoretical red curve (that fits infected worms) to yield the green one (that fits antibiotic-treated worms) was achieved by turning down a single parameter, the co-ageing constant, which quantifies the impact of an antagonist. All other parameters (see the electronic supplementary material) were kept constant.

We keep track of the cause of malfunction of the nodes so that we can tell the cause of death of each network. This will later enable us to fit the data of Zhao *et al.* [24] who were able to resolve between worms dying of old age (intrinsic damage) versus infection (co-ageing damage). This way, our theory will be able to fit not only all-cause hazard curves but also, simultaneously, the cause-specific hazard curves (figure 3). Furthermore, we will even be able to fit the antibiotic-treated worms (which do not co-age) and non-treated worms (which do co-age) using the same parameter values, except for simply turning down the bacterial impact, $C_{B}$.

The number of functional and two types of dysfunctional nodes (owing to intrinsic ageing and co-ageing) at time t are denoted by f(t), $n_{s}(t)$ and $n_{c}(t)$. When a network collapses, we record whether its failure was mostly owing to intrinsic damage (if $n_{s}>n_{c}$) or co-ageing damage (if $n_{s}<n_{c}$), which defines in our model, the cause of death.

Once the entire cohort dies, the all-cause hazard curves (probability of death) μ(t)=[S(t)−S(t+1)]/S(t) are obtained in terms of the number of survivors S(t) at t. Following the framework of competing risks analysis, death owing to co-ageing and intrinsic damage are two mutually exclusive, competing events—each individual can only die from one of these two causes. The cause-specific hazard curves are then defined as, $\mu_{i}(t)=d_{i}(t)/S(t)$, where $d_{s}(t)$ and $d_{c}(t)$ represent the number of individuals who die from intrinsic damage and co-ageing damage, respectively, at time t. In this formulation, the all-cause hazard curve is simply the sum of the cause-specific hazard curves. For a more detailed discussion of competing risks analysis and cause-specific hazard rates, see [27–29].

## 3. Results

### 3.1. Overview

In our simulations, the fraction of nodes alive, f(t), declines gradually, followed by a sudden collapse (figure 2, insets). When we compare f(t) for species ageing in solitude versus co-ageing with an antagonist, we see that the latter exhibits a sharp change in slope coinciding with the average lifespan of its antagonist (figure 2, vertical dashed line).

Figure 2 depicts the hazard curves for species A with (left) and without (right) its co-ageing antagonist. The insets show the functional decline of 50 randomly chosen A individuals. The vertical dashed line marks $\tau_{B}$, the mean lifespan of B. In the left inset, nearly all curves exhibit a sharp change in slope after the expected lifespan of the antagonist, as co-ageing damage diminishes after this time.

In figure 2, the difference between the two hazard curves is solely owing to the value of the co-ageing parameter $C_{A}$, which causes the hazard curve to have a ‘bump’ around $\tau_{B}$, the mean life span of the antagonist. Here, μ(t) increases from birth until $\tau_{B}$ owing to the combined effect of the intrinsic and co-ageing damage, starts to decrease as the antagonists perish (owing to intrinsic and co-ageing damage of their own), followed by, again, a late-life increase in mortality owing to the system’s intrinsic damage.

The hazard curve on the right was run with the same parameters except for $C_{A}$ set to zero. With this, the bump vanishes, and we recover ageing trajectories observed more typically, as in Vural *et al*. [7].

### 3.2. Disentangling the two types of ageing in worms

Recently, Zhao *et al*. [24] measured the hazard curves of a *C. elegans* population coexisting with their pathogen *E. coli* and those that were treated with antibiotics so that they age intrinsically. They also recorded the cause of death of the worms through necropsy analysis, by observing severe swelling of the posterior pharyngeal bulb in individuals who died earlier in life owing to infection and atrophy of the same region in those who died later owing to other causes (in our interpretation, owing to intrinsic damage). During early life, *C. elegans* exhibits rapid pharyngeal pumping that can damage the pharyngeal cuticle, making individuals more susceptible to *E. coli* invasion. However, owing to heterogeneity in the population, some individuals are able to resist this invasion, while others get colonized and die. This way, Zhao *et al*. [24] were able to report cause-specific hazard curves as well as all-cause ones. For the details of population heterogeneity in the context of *C. elegans*, see Le Cunff *et al*. [30,31].

In figure 3, we fit the empirical measurements of Zhao *et al*. [24] using our theory. In figure 3*a*, we show the cause-specific hazard curves, where $\mu_{i}(t)=d_{i}(t)/S(t)$, with $d_{c}(t)$ representing the number of individuals who died owing to co-ageing and $d_{i}(t)$ representing those who died from intrinsic damage. S(t) denotes the number of surviving individuals at time t. In figure 3*b*, we fit the all-cause hazard curves, both in the absence (by summing the cause-specific hazard curves from figure 3*a*) and in the presence of antibiotics. We should emphasize that we fit all data using a single combination of parameters, except for reducing the parameter that quantifies the bacteria-inflicted damage ($C_{A}=0.023$ to $C_{A}=0.013$) to account for the presence of antibiotics in some of the experiments.

In figure 3*a*, we observe that co-ageing-induced mortality is more pronounced in the early stages of life. As a significant portion of infected individuals are eliminated, an increase in intrinsic-damage-induced mortality emerges where co-ageing mortality is at its peak. This increase eventually plateaus.

In summary, our model generates a non-monotonic all-cause hazard curve as the sum of two cause-specific hazard curves: one is a monotonically increasing curve resulting from intrinsic damage accumulation in the organism, while the other is a non-monotonic curve caused by co-ageing damage.

### 3.3. Suppression in trees as a form of co-ageing

There are numerous factors contributing to tree mortality, ranging from fires, winds, drought and predation [32–35] to less apparent factors such as competition from other trees, pathogens and insects [20,22,36–39]. Some damaging factors occur uniformly throughout the lifespan of the tree, while others are age-dependent [23]. Our primary focus here will be on the ‘thinning’ or so-called ‘stem-exclusion’ stage, where one of the most dominant mortality drivers is the competition for other trees for resources [20–23]. Suppression is a mortality factor that poses a lethal threat not only on its own but also by exacerbating other factors. This means that even when competition itself is not the direct cause of death, it can potentially intensify the adverse impacts on trees resulting from factors like climate change [40].

Lutz & Halpern [20] collected 22 years of tree growth and mortality data from early successional forests. The mortality curves they measured exhibited a non-monotonic pattern for all six tree species under investigation. This study provided empirical evidence that suppression stands as the most prevalent form of mortality during the initial phases of stand development.

**Figure 4.**
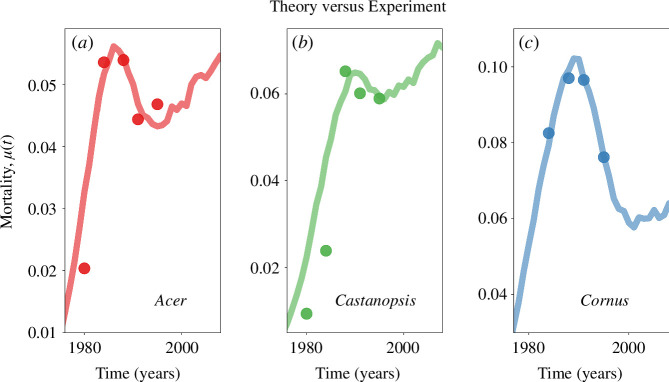
Co-ageing of three tree species with their competitors (dots), fitted to co-ageing networks (curves)**.** The non-monotonic hazard curves of(*a*) *A. macrophyllum*, (*b*) *C. chrysophylla* and (*c*) *C. nuttallii* [20], characterized by a decrease in mortality rates around the mid-stages of the observed period. Our model successfully replicates this trend by showing a similar decline during the intermediate stages of thinning. While trees are affected by the cumulative competition arising from the neighbouring trees, our model simplifies these complex relationships by reducing all competitors to a single representative system. For model parameters, see the electronic supplementary material.

In figure 4, we compare our theoretical model to three of their datasets, fitting for *Acer macrophyllum*, *Castanopsis chrysophylla* and *Cornus nuttallii*. The mortality values reported by Lutz & Halpern [20] were defined as $m(t_{i})=1-(S(t_{f})/S(t_{i}){)}^{1/\Delta t}$, where m(t) is the mortality, S(t) is the number of alive individuals and $\Delta t=t_{f}-t_{i}$ with $t_{i}$ and $t_{f}$ corresponding to time of initial and final measurement times, respectively. In our simulations, each Δt=1, thus the formula reduces to our definition for mortality calculation μ(t)=[S(t)−S(t+1)]/S(t). For a discussion of definitions of mortality in this context, see Sheil *et al*. [41].

### 3.4. Co-ageing in non-biological complex systems: chess games

We expect to see the demographic signatures of co-ageing whenever two complex systems with high interdependency damage one another, regardless of whether they are biological or not. Examples would include rivalling companies, armies, social organizations, sports teams and countries.

Here, we test this claim by turning to a simple model system, chess, and leave the analysis of the other complex systems to future studies.

In chess, pieces organize to protect each other, forming dynamic networks. As opponents attack each others’ network of defences, they should co-age in a similar way to their biological analogues and our simulated interdependency networks. These games provide us with a highly controlled and tunable experimental environment where the relative strength of players can be adjusted, health status can be easily monitored as a function of time and population-scale measurements can be collected rapidly.

To obtain hazard curves in high resolution, we examine two datasets, one is from the Free Internet Chess Server (FICS) and the other one is gathered by us. The FICS dataset spans across games played between 2015 and 2019 on their server. The way FICS assign a rank to each player is by a calculation of an “Elo score” through games they have played: A higher Elo score signifies a greater likelihood of winning and the rating deviation (RD) indicates the accuracy of the reported Elo score.

FICS employs the Glicko rating system [42], a commonly adopted method to estimate player strength in zero-sum games. The 95% confidence interval of the Elo rating is calculated as ±1.96 RD. This framework also enables the computation of the expected outcome of the game for a player i competing against player j, $E_{ij}$, as:


(3.1) $$E_{ij}=\frac{1}{1+10^{-g(\sqrt{{RD}_{i}^{2}+{RD}_{j}^{2}})(r_{i}-r_{j})/400}},$$

where $g(x)=1/\sqrt{1+3q^{2}(x^{2})/\pi^{2}}$, q=ln⁡10/400, $r_{i}$ and $r_{j}$ are the Elo ratings of the players [42]. Outcome of a game for a player can take either 1, 1/2 or 0 corresponding to win, draw or loss. A player with higher Elo is expected to be able to inflict more damage per move and receive less. Thus we associate the expected outcome of the game (which is a function of Elo ratings and RDs) with our co-ageing parameters $C_{A,B}$. To maintain consistency across games, we focus on those where $E_{ij}$ falls within a pre-defined interval (e.g., between 0.7 and 0.8), ensuring minimal fluctuation in expected game outcomes among the examined games. Moreover, we maintain a constant allocated time of 5 min per game and exclude the games that resulted in a draw or forfeit.

For the second dataset, we facilitate games between pairs of *Stockfish* chess engines and record the lifespan of players. One of the crucial parameters characterizing the strength of a chess engine is its thinking depth D, which quantifies the number of moves the engine can anticipate while calculating the best possible move. An engine with higher depth is able to inflict more damage per move and receive less. In this case, we associate the thinking depth with our co-ageing parameters $C_{A,B}$.

There are a number of subtle differences between a chess army and an organism. In chess, when one party loses, the game ends for both the winner and loser, whereas in biology, the winner continues to live, and better so, in the absence of an adversary. Thus, to analyse chess games and biological species on equal footing, we let the winning side continue to ‘live’ after the game ends. We adopt this convention in our simulations too, i.e. when one network kills the other network, we let the winner continue to live instead of ending the simulations. When calculating mortality for the chess games, we follow the same formula given as $\mu_{i}(t)=[S_{i}(t)-S_{i}(t+1)]/S_{i}(t)$, where $S_{i}(t)$ represents the number of chess players still playing at time t or already won the game, with i indicating whether the player is white or black.

Lastly, unlike biological organisms, chess armies do not experience any other damage apart from that inflicted by their opponents, $d_{A}=d_{B}=0$; they do not start with any ‘prenatal’ damage, $f_{A}(0)=f_{B}(0)=1$; cannot repair themselves, $r_{A}=r_{B}=0$; and their sizes are precisely equal $N_{A}=N_{B}$. Moreover, if we were to vary the strength difference between human players (via tuning $E_{ij}$) or thinking depth difference between machine players, to update our theoretical fit, we only allow ourselves to modify the co-ageing damage parameters $C_{A,B}$. For example, if the expected outcome changes in favour of whites, we would lower $C_{A}$ (damage received by A) while increasing $C_{B}$ (damage received by B), keeping all other parameters constant.

**Figure 5.**
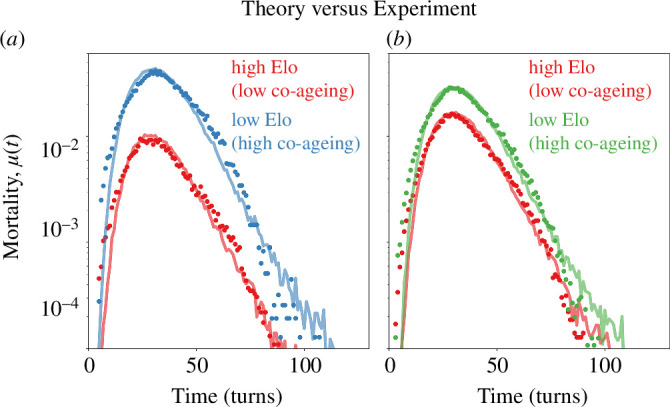
Co-ageing in chess games. (*a*) We collected the failure statistics of chess games with the expected outcome falling between 0.70 and 0.80 for whites with 5 min allocated time per game. We achieved fits to our theoretical model by setting d=0 (no damage source other than that of the antagonist), f(0)=1 (no prenatal damage), r=0 (no repair), which are reasonable assumptions for a chess game. (*b*) As expected outcome of the games for whites ranges from 0.55 to 0.65 hazard rate of the whites elevates, and the one for black decreases. To fit the changed hazard curves, we modified only the co-ageing parameters in our simulations, such that the white-representing network receives more damage from the black and the black-representing network receives less damage from the white. To represent different strategies in each chess game, we randomized the connectivity structure while keeping the number of nodes N constant. The threshold of death is chosen at 15% in (*a*,*b*), and the other parameters were kept constant (see the electronic supplementary material for further discussion about the threshold).

Despite these severe parametric restrictions, the model fits the FICS data quite well (figure 5). Figure 5*a* illustrates the likelihood of losing the game for both the empirical data (dots) and the network simulations (curves), where $E_{wb}\in [0.7,0.8]$ in favour of whites. Meanwhile, in figure 5*b*, the relative strength difference is reduced to $E_{wb}\in [0.55,0.65]$. Notably, there are only two free parameters, $C_{A}$ and $C_{B}$, consistent with the constraints outlined above: moving from figure 5*a*,*b*, the expected outcome of the game becomes more balanced, indicating a decrease in the likelihood of whites winning. Accordingly, we fitted the data by decreasing the co-ageing damage inflicted by white while increasing it for black.

Presumably, chess games have less interdependent components compared to worms and trees, and vital/core pieces constitute a greater percentage of the network. As such, to fit the chess data, we had to significantly reduce the size of the networks and raise the death threshold ($\phi_{T}=0.15$).

On a related note, in our simulations, we have observed that small self-sufficient sub-networks (typically, dense cliques) linger even after the rest of the network dies, blurring the line between life and death. Such structures are less likely to occur in large networks, but they influence (especially in late-life) the mortality statistics of small networks: the smaller the network size, the more dependent late-life hazard trajectories are, on the choice of death threshold. See the electronic supplementary material for a detailed discussion on the network death threshold and for a fit to data with a different choice of threshold, $\phi_{T}=0.10$.

**Figure 6.**
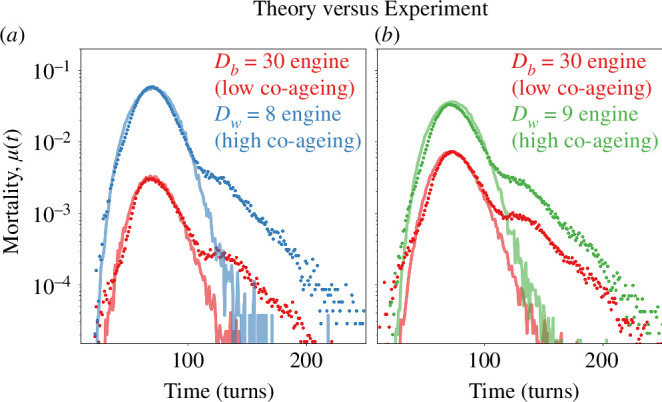
Co-ageing in chess engines. (*a*) We collected the failure statistics of two chess engines playing 400 000 games against each other (dots), where whites have thinking depth, $D_{w}=8$ and the blacks, $D_{b}=30$. We achieved the fits (curves) to our model by setting d=0 (no damage source other than that of the antagonist), f(0)=1 (no prenatal damage), r=0 (no repair), which are reasonable assumptions for a chess game. (*b*) The black’s hazard rate elevates, when its opponent white has a higher thinking depth of $D_{w}=9$. For both (*a,b*), engines are limited to 0.01 s per move. To fit the new hazard curves on (*b*), we modified only the co-ageing parameters in our simulations. Each of the simulations are carried out with the same networks to ensure consistency with the chess games played by the same engines. Our model fits the former dataset very well, but exhibits an interesting departure for machine players. It is interesting that human and machine players exhibit qualitatively different hazard curves, for which we do not have a decisive explanation.

In figure 6, we fit our network simulations to data of games played by chess engines. Here, figure 6*a* displays the probability of losing the game as a function of turns, while the chess engine of depth $D_{w}=8$ (white) plays against one of depth $D_{b}=30$ (black). Figure 6*b* displays the mortality rates of same engine playing blacks with $D_{b}=30$ against an engine playing whites with $D_{w}=9$. As the depth of the engine playing whites increases from figure 6*a*,*b*, we observe a corresponding rise in the mortality rate of blacks, coupled with a decrease in the mortality rate of whites. For both figure 6*a*,*b*, we only consider chess games concluded with the checkmate of either side and exclude any draws. We use fit using only two free parameters, $C_{A}$ and $C_{B}$, considering the self-imposed restrictions.

Curiously, the late-life mortality characteristics of chess engine hazard rates are different from that of human players. In figure 5, we see an abrupt shift in the rate of decline of the mortality rate. While we cannot explain this difference decisively, we discuss possible reasons for this interesting difference in the electronic supplementary material.

### 3.5. A closer look at the effects of individual simulation parameters

**Figure 7.**
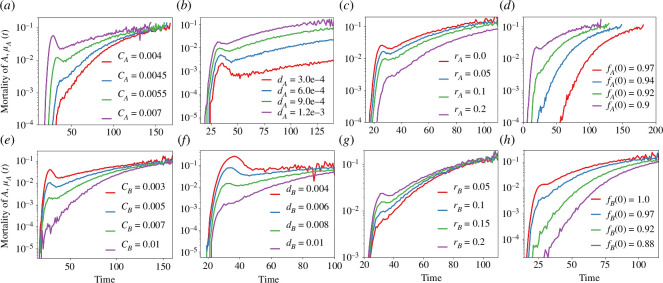
The effect of system parameters on hazard curves. (*a*) Increasing the co-ageing constant $C_{A}$ results in the formation of a ‘kink’ followed by a ‘bump’, which corresponds to a local maximum and increased probability of death in early life owing to co-ageing. (*b*) Increasing the intrinsic damage rate $d_{A}$ causes a shift in the hazard curve upwards. (*c*) As the repair rate, $r_{A}$, increases, both co-ageing and intrinsic ageing effects dissipate. (*d*) Decreasing prenatal damage, $f_{A}(0)$, leads to an increase in mortality and highlights the co-ageing effects with the formation of a ‘bump’. (*e*) Increasing $C_{B}$ results in the dissipation of the bump owing to increased probability of death of B in early life. (*f*) Increasing the intrinsic ageing rate $d_{B}$ causes a shift in the hazard curve downwards. (*g*) As the repair rate, $r_{B}$ increases, both co-ageing and intrinsic ageing effects are emphasized. (*h*) Decreasing prenatal damage, $f_{B}(0)$, leads to a decrease in mortality and highlights the co-ageing effects with the dissipation of the ‘bump’. Details of the parameters for each of the curves are provided in the electronic supplementary material.

Figure 7*a*–*d* describes the influence of system parameters on the hazard curves of A. The co-ageing constant emerges as a crucial determinant of the qualitative shape of the hazard curve, with increasing values resulting first in a ‘kink’ followed by a ‘bump’, as shown in figure 7*a*. This effect can be attributed to A becoming more adversely affected by B, causing a local maximum early on. Moreover, we observe the curve shifting to the left and reaching a plateau earlier as $C_{A}$ increases. In figure 7*b*, we observe that the increase in the intrinsic ageing rate $d_{A}$ of the network causes the hazard curve to shift upwards, including the bump without changing its x coordinate. This shift can be attributed to the increasing effect of intrinsic ageing compared to co-ageing with the increase of $d_{A}$. By contrast, increasing the repair rate of the networks A diminishes the effect of both ageing types. As shown in figure 7*c*, the ‘bump’ turns into a ‘kink’ and eventually disappears completely, resulting in a reduced plateau. In figure 7*d*, we examine the effect of prenatal damage, quantified by the initial number of functional nodes f(t=0). An increase in prenatal damage highlights the co-ageing effects on the system, leading to the appearance of a ‘bump’ with emphasized co-ageing for lower values of f(t=0).

Figure 7*e*–*h* presents the influence of system parameters of type B networks on the hazard curves of type A networks. In figure 7*e*, as the co-ageing constant $C_{B}$ increases, type B networks exhibit a diminished co-ageing effect on type A networks. Consequently, the previously observed bump in the hazard curve diminishes and eventually disappears as this parameter increases. Notably, unlike the case of changing $C_{A}$, the position of the bump remains unchanged. In figure 7*f*, we observe that an increase in the intrinsic ageing rate $d_{B}$ of network B causes the bump to vanish without affecting the saturation value of mortality. On the other hand, increasing the repair rate of network B intensifies the bump without altering the initial and saturation mortalities. Figure 7*g* demonstrates the transformation of the ‘bump’ into a ‘kink’, which eventually vanishes with decreasing repair rate of network B. Figure 7*h* shows that an increase in prenatal damage diminishes the co-ageing effects on the type A mortality. This shift results in postponing the initial appearance of mortality and shifting the curve to the right.

## 4. Conclusion

We introduced the concept of co-ageing as the mechanism behind the anomalous features observed in the hazard curve of some organisms, which can be explained by neither evolutionary theories nor simpler reliability and interdependence network theories. We demonstrated, however, that incorporating the notion of co-ageing into the interdependence picture, can account for these anomalies.

To provide empirical support for our thesis, we fitted three very different co-ageing systems into our model. We were able to go beyond one fit per system since our framework could make quantitative sense of experimental variables within individual datasets, such as treating the worms with antibiotics, cause-specific deaths, varying the relative strength of players/engines or varying the species of trees. Our framework was also able to go beyond fitting just all-cause hazard curves. Instead, we were able to fit independently measurable cause-specific components that add up to build the all-cause hazard curve.

The bumps and kinks observed in the hazard curves across diverse living and non-living complex systems are a potential signal for co-ageing. As such, we propose that ageing demographics can be used as a kind of microscope with which ecological interactions can be probed.

We end with a reminder that our model, like all models, is a simplified representation of a phenomenon, which in reality is extremely complex: we have not taken into account the time-dependent nature of interdependency networks (chess pieces move and immune systems adapt), the heterogeneity of nodes (not all cells/pieces have the same value and impact), the strategic (non-random) nature of attacks and defences and potentially many other interesting aspects of co-ageing. Nevertheless, and despite these handicaps, we were able to unite within one theoretical framework, sickly worms, competing trees and chess battles.

## Data Availability

Data and relevant code for this research work are stored in GitHub: https://github.com/cagatayeskin/Co-Aging and have been archived within the Zenodo repository [43]. Supplementary material is available online [44].
